# Interrater Reliability of the Modified Tuck Jump Assessment Scored by Physical Therapy Students

**DOI:** 10.7759/cureus.116242

**Published:** 2026-09-14

**Authors:** Makoto Saito

**Affiliations:** 1 Department of Rehabilitation, Aichi Medical College for Physical and Occupational Therapy, Kiyosu, JPN

**Keywords:** anterior cruciate ligament, female athletes, interrater reliability, modified tuck jump assessment, movement analysis, physical therapy students, sports injury screening

## Abstract

Background: The Modified Tuck Jump Assessment (TJA) is a field-based, video-based tool used to evaluate movement faults relevant to anterior cruciate ligament (ACL) injury mechanisms. Although its reliability has been examined among experienced evaluators, its reliability when scored by novice evaluators remains unclear. This study investigated the interrater reliability of the Modified TJA when scored by physical therapy students without clinical experience.

Methods: This cross-sectional reliability study included 48 female high school soccer and rugby athletes from Japan. Participants performed the 10-second Modified TJA, comprising 10 movement-quality items scored from 0 to 2. Two final-year physical therapy students without clinical experience independently evaluated frontal and sagittal video recordings. Item-level agreement was assessed primarily using linear-weighted Cohen’s kappa, with 95% confidence intervals estimated using a participant-level bootstrap with 20,000 resamples. Unweighted kappa and exact percentage agreement were calculated as secondary measures. Total-score reliability was assessed using ICC(2,1), a two-way random-effects model for single measurements and absolute agreement.

Results: The total score demonstrated excellent interrater reliability (ICC(2,1) = 0.868; 95% CI, 0.775-0.924). Linear-weighted kappa coefficients for individual items ranged from 0.30 to 0.78; four items demonstrated substantial agreement, four demonstrated moderate agreement, and two demonstrated fair agreement. Six of the 10 items did not demonstrate substantial agreement, and several confidence intervals were wide. Exact agreement occurred in 356 of 480 item-level comparisons (74.17%) and ranged from 58.33% to 85.42% across items.

Conclusions: Two physical therapy students without clinical experience demonstrated excellent interrater reliability for the total Modified TJA score, whereas item-level agreement was heterogeneous and sometimes imprecise. These findings indicate consistency between the two student raters but do not establish scoring accuracy because their ratings were not compared with those of experienced evaluators or a criterion standard. Item-level results should therefore be interpreted alongside the total score, and standardized training should be evaluated before students use the Modified TJA independently.

## Introduction

Anterior cruciate ligament (ACL) injury is a major concern in sports because of its effects on athletic participation, long-term health, and cost [1,2]. Female athletes have a higher incidence than male athletes, and many ACL injuries occur without contact during landing or rapid directional changes [3-5]. Potentially modifiable neuromuscular patterns associated with these mechanisms include excessive knee valgus and poor trunk control [6,7].

Three-dimensional motion analysis provides detailed kinematic and kinetic measurements, but its cost, required expertise, calibration, and processing time limit routine use in schools, clinics, and field-based screening [8]. The Tuck Jump Assessment (TJA) was selected because it requires minimal equipment and evaluates repeated maximal jumps over 10 s rather than a single squat or drop-landing task. Its repeated, high-intensity format may reveal movement faults that emerge with fatigue or loss of feed-forward control. The 10 criteria capture manifestations related to ligament dominance (excessive frontal-plane knee motion), quadriceps dominance (a relatively stiff landing strategy), leg dominance (side-to-side asymmetry), and trunk dominance (inadequate trunk control), as well as deterioration in technique and reactive jumping performance [9]. The Modified TJA extends the original binary scale by grading each fault as absent, small, or large (0-2), allowing the magnitude of observed deficits to be represented [9].

Previous work demonstrated high reliability when the Modified TJA was scored by experienced strength and conditioning coaches [9], but agreement among novice evaluators is uncertain. The objective of this study was therefore to determine whether two physical therapy students without clinical experience could obtain acceptable interrater reliability for the total score and individual items.

## Materials and methods

Participants

This study involved 48 female high school athletes from Japan, comprising 24 soccer players and 24 rugby players. Individuals were excluded if they were undergoing medical treatment for pain or illness at the outset of the study, were unable to perform at their usual level, or were non-athletes such as team managers. Participant demographics are presented in Table 1.

**Table 1:** Characteristics of the study participants Values are presented as mean ± standard deviation (SD).

Characteristic	Mean ± SD
Age (years)	16.31 ± 0.97
Years of sports experience (years)	4.67 ± 2.20
Height (cm)	158.32 ± 5.89
Weight (kg)	55.04 ± 7.18
Body mass index (kg/m^2^)	21.94 ± 2.51

One week before data collection, the researchers visited each high school and provided verbal and written explanations of the study. The study protocol was approved by the Research Ethics Committee of Aichi Medical College for Physical and Occupational Therapy (Approval No. 24013). Participants were informed of the procedures, potential risks, safety measures, voluntary nature of participation, and right to withdraw without consequences for club activities. Written informed consent was obtained from all participants. Separate parental consent was not obtained; the participant-consent procedure was reviewed and approved by the ethics committee for this study.

No a priori sample size calculation was performed. As a post hoc benchmark, application of the method described by Walter et al. [10] indicated that 39 participants would be required to distinguish an ICC of 0.80 from 0.60 with two raters, a one-sided alpha of 0.05, and 80% power. The final sample of 48 participants exceeded this benchmark.

Study design

This cross-sectional study aimed to evaluate the interrater reliability of the Modified TJA, a screening tool used to identify neuromuscular deficits associated with ACL injury in female soccer and rugby athletes.

Measurement procedure

Participants were given an explanation of the procedure through a live demonstration by the researcher. The instructions included the following points: feet should be positioned shoulder-width apart, knees should be raised so that the thighs are parallel to the ground, both feet must land simultaneously at the same location, jumps should be confined within a designated box (41 cm long × 35 cm wide), and jumps should be performed continuously for 10 s without pausing. After confirming that participants understood the test requirements, they completed a warm-up followed by up to two practice trials of the Modified TJA. The Modified TJA was then conducted in a designated space and recorded for 10 s using two video cameras: one placed 3 m from the participant to capture the sagittal view and the other placed 3 m from the participant to capture the frontal view.

Basic demographic and background data, including age, height, weight, and years of experience in their respective sports, were also collected.

Modified TJA scoring and analysis

Two final-year physical therapy students served as evaluators. Both had completed foundational coursework in anatomy, physiology, and movement-related subjects but had no clinical or field-based practical experience. Before scoring, a licensed physical therapist conducted a single verbal briefing based on the published Modified TJA criteria [9]. The briefing reviewed the 10 items, the 0-2 response scale, and the rule that a technique fault had to occur on at least two repetitions. The duration of the briefing was not recorded. No practice scoring of actual footage, feedback session, or calibration exercise was provided. Each evaluator then scored the frontal and sagittal recordings independently, without access to the other evaluator's scores. Playback speed and the use of slow-motion or frame-by-frame review were not standardized. The Modified TJA includes 10 items rated from 0 to 2 according to the published scoring criteria [9]. Lower total scores indicate better performance and fewer observed movement faults.

Statistical analysis

The total-score analysis and descriptive statistics were performed using IBM SPSS Statistics for Windows, Version 29.0.1.0 (Released 2023; IBM Corp., Armonk, NY, USA). Because the item scores were ordinal (0-2), linear-weighted Cohen's kappa was used as the primary measure of item-level agreement. Ninety-five percent confidence intervals were estimated using a participant-level bootstrap with 20,000 resamples in Python 3.12.13 (Python Software Foundation, Fredericksburg, VA, USA).

Unweighted Cohen's kappa and exact percentage agreement were retained as secondary measures for comparison with earlier Modified TJA reliability reports. The total score was assessed using ICC(2,1), a two-way random-effects model based on single measurements and absolute agreement. Kappa values were interpreted as follows: <0, no agreement; 0.00-0.20, slight; >0.20-0.40, fair; >0.40-0.60, moderate; >0.60-0.80, substantial; and >0.80-1.00, almost perfect [11]. ICC values were interpreted as follows: >0.75, excellent; >0.60-0.75, good; 0.40-0.60, fair; and <0.40, poor [12].

## Results

Interrater reliability of the Modified TJA total score was examined using a two-way random-effects model with absolute agreement. The single-measure ICC(2,1) was 0.868 (95% CI, 0.775-0.924), indicating excellent interrater reliability. The corresponding F statistic was F(47, 47) = 13.866 (p < 0.001).

For the individual items, linear-weighted kappa coefficients ranged from 0.30 to 0.78. Four items demonstrated substantial agreement: “Thighs Not Equal Side-to-Side (Asymmetry)” (Item 3), “Does Not Land in Same Footprint” (Item 7), “Excessive Landing Noise” (Item 8), and “Technique Declines Prior to 10 Seconds” (Item 10). Four items demonstrated moderate agreement: “Lower Extremity Valgus at Landing” (Item 1), “Thighs Do Not Reach Parallel (Peak of Jump)” (Item 2), “Foot Placement Not Parallel” (Item 5), and “Pause Between Jumps” (Item 9). “Foot Placement Not Shoulder-Width Apart” (Item 4) and “Foot Contact Timing Not Equal” (Item 6) demonstrated fair agreement. Unweighted kappa coefficients ranged from 0.28 to 0.68 (Table 2). Across all 10 items, exact agreement occurred in 356 of 480 item-level comparisons (74.17%).

**Table 2:** Item-level interrater agreement for the Modified Tuck Jump Assessment Linear-weighted kappa was the primary item-level measure. Confidence intervals were estimated using a participant-level percentile bootstrap with 20,000 resamples.

Criteria	Unweighted κ	Linear-weighted κ (95% CI)	Exact agreement, n (%)
1. Knee valgus at landing	0.49	0.53 (0.32-0.72)	32 (66.67)
2. Thighs below parallel	0.42	0.55 (0.36-0.70)	30 (62.50)
3. Unequal thigh height	0.68	0.78 (0.59-0.91)	41 (85.42)
4. Foot width	0.28	0.32 (0.06-0.55)	28 (58.33)
5. Front-back foot alignment	0.53	0.58 (0.37-0.76)	35 (72.92)
6. Foot-contact timing	0.34	0.30 (0.04-0.57)	37 (77.08)
7. Does not land in same footprint	0.68	0.69 (0.50-0.87)	40 (83.33)
8. Excessive landing noise	0.60	0.69 (0.48-0.85)	39 (81.25)
9. Pause between jumps	0.43	0.45 (0.08-0.74)	39 (81.25)
10. Technique declines prior to 10 seconds	0.56	0.63 (0.41-0.80)	35 (72.92)

## Discussion

This study examined the interrater reliability of the Modified TJA when scored by physical therapy students without clinical experience. The total score showed excellent interrater reliability (ICC(2,1) = 0.868; 95% CI, 0.775-0.924). In contrast, item-level linear-weighted kappa coefficients ranged from 0.30 to 0.78, with agreement ranging from fair to substantial. For interpretation, weighted kappa values of 0.60 or lower were considered to indicate less than substantial agreement, based on the classification proposed by Landis and Koch [11]. Confidence intervals were also wide for several items. The principal finding is therefore strong agreement for the aggregate score alongside heterogeneous and often insufficiently precise agreement for specific movement faults.

This distinction is clinically important because identical total scores can arise from different combinations of item scores. A high total-score ICC may therefore mask disagreement about the particular movement deficits the assessment is intended to identify. Item-level estimates and their confidence intervals should be considered alongside the total score rather than treating the aggregate ICC as evidence that all criteria can be scored reliably.

For Items 6 and 9, the relatively low linear-weighted kappa values (0.30 and 0.45, respectively) may be partly explained by limited score variability, despite exact agreement for 37 of 48 participants (77.08%) and 39 of 48 participants (81.25%), respectively. Notably, 33 participants received a score of 0 from both raters for Item 6, and 34 participants received a score of 0 from both raters for Item 9. This limited variability may have been influenced by the homogeneity of the participant group, which consisted of athletes of similar ages and backgrounds.

The lower agreement for Items 1, 2, 4, 5, 6, and 9 may reflect the evaluators' limited experience in observing rapid multiplanar movement. In the Landing Error Scoring System, novice raters can achieve agreement with experts when standardized criteria and training are provided [8]. Conversely, research using standardized TJA rater training still found that reliability for the total score was higher than agreement for many individual technique faults [13]. Together, these findings suggest that novice status alone does not preclude reliable scoring, but item-specific anchors, example videos, and calibration are important when visual judgments involve subtle spatial or temporal distinctions.

The raters in the present study received a verbal explanation of the criteria but no practice with actual footage or feedback before scoring. Items requiring judgments of foot width, knee height, or contact timing may also have been affected by two-dimensional viewing, which provides no direct depth information. In addition, camera resolution and frame rate were not retained in the study records, and playback speed and slow-motion review were not standardized. These technical and procedural factors may have made brief or subtle movement differences more difficult to judge and should be specified and standardized in future protocols.

Standardized training that includes item-specific examples and calibrated practice may improve agreement, but this was not tested in the present study. Similarly, slow-motion or frame-by-frame review is a plausible strategy for criteria requiring precise spatial or temporal judgments, but its effect should be evaluated prospectively rather than assumed from the current data. The present results therefore support further development and testing of a standardized novice-rater protocol, not an immediate recommendation that students independently conduct clinical screening.

This study has several limitations. First, only two physical therapy students from one educational program served as evaluators, limiting generalizability to other students, programs, and levels of training. The sample comprised 48 athletes; this exceeded the post hoc benchmark for the total-score ICC, but larger and more diverse samples would provide greater precision for item-level estimates. Second, participants were limited to female high school soccer and rugby athletes, so the findings may not generalize to other ages, sexes, sports, or performance levels. Third, the student ratings were not compared with ratings from experienced evaluators or with three-dimensional motion analysis. Consequently, high agreement indicates consistency between the two students but does not establish scoring accuracy; both raters could agree while sharing the same systematic error. Fourth, aggregation may conceal item-level disagreement because the same total can arise from different fault profiles. Fifth, the duration of rater instruction and camera specifications were not retained, and playback procedures were not standardized. Finally, the study evaluated interrater reliability, not the criterion or predictive validity of the Modified TJA for ACL injury risk.

Future studies should include larger and more diverse rater samples, expert or criterion-standard comparisons, prespecified video specifications, and standardized training with calibrated examples. Randomized or sequential studies should test whether practical training and slow-motion or frame-by-frame review improve item-level reliability and accuracy. Further work should also examine criterion and predictive validity before Modified TJA scores are interpreted as indicators of ACL injury risk.

## Conclusions

Two physical therapy students without clinical experience demonstrated excellent interrater reliability for the total Modified TJA score, whereas item-level agreement was heterogeneous, with 6 of the 10 criteria showing less than substantial agreement and several estimates having wide confidence intervals. These findings indicate consistency between the two student raters but do not establish scoring accuracy or agreement with experienced evaluators. Further studies should examine item-level reliability, standardized rater training, and criterion validity in larger and more diverse samples. The potential benefit of slow-motion review should also be evaluated prospectively.
